# Evaluation of the Physical Behavior, Biocompatibility, and Biosafety of AMCOP Elastodontic Appliances: A Prospective Observational Study

**DOI:** 10.3390/ma19214717

**Published:** 2026-11-06

**Authors:** Angelo Michele Inchingolo, Grazia Marinelli, Pierluigi Marotti, Filippo Cardarelli, Francesco Magistro, Daniela Di Venere, Francesco Inchingolo, Alessio Danilo Inchingolo, Gianna Dipalma

**Affiliations:** 1 Department of Interdisciplinary Medicine, University of Bari “Aldo Moro”, 70124 Bari, Italy; angelomichele.inchingolo@uniba.it (A.M.I.); graziamarinelli@live.it (G.M.); pierluigi.marotti@uniba.it (P.M.); drfilippocardarelli@libero.it (F.C.); orthoproteclab@gmail.com (F.M.); daniela.divenere@uniba.it (D.D.V.); alessiodanilo.inchingolo@uniba.it (A.D.I.); gianna.dipalma@uniba.it (G.D.); 2 Department of Medicine and Surgery, University of Perugia, 06132 Perugia, Italy; 3 Department of Biomedical, Surgical and Dental Sciences, Milan University, 20122 Milan, Italy

**Keywords:** elastodontics, AMCOP, functional orthodontics, elastodontic appliances, biomaterials, biocompatibility, biosafety, thermoplastic elastomers, interceptive orthodontics

## Abstract

**Introduction:** Elastodontic appliances have become increasingly popular in interceptive orthodontics because they simultaneously address dentoalveolar discrepancies and orofacial dysfunctions while promoting physiological craniofacial growth. Their long-term clinical effectiveness, however, depends not only on treatment protocols but also on the physical stability, biocompatibility, and biosafety of the materials from which they are manufactured. Although laboratory testing and regulatory certifications provide evidence regarding material safety, limited clinical data are available concerning the behavior of these appliances after prolonged intraoral use. **Objective:** The present study aimed to evaluate the physical behavior, biocompatibility, and biosafety of AMCOP elastodontic appliances after six months of clinical use through a prospective observational research. **Materials and Methods:** Ten patients undergoing interceptive orthodontic treatment with AMCOP elastodontic appliances were prospectively monitored for six months. All participants followed an identical clinical protocol consisting of appliance wear for at least one hour during daytime and throughout the night, combined with standardized daily cleaning using liquid Marseille soap. Clinical evaluation included assessment of surface integrity, dimensional stability, maintenance of appliance morphology, elastic behavior, surface wear, permanent deformation, fractures, and overall clinical functionality. Biological outcomes included oral mucosal tolerance, allergic reactions, irritation, patient-reported comfort assessed using a Visual Analogue Scale (VAS), treatment compliance, transient hypersalivation, and overall patient satisfaction. **Results:** After six months, no visible dimensional changes were observed on clinical examination in any of the ten appliances. No fractures or clinically apparent structural discontinuities were detected. Minor superficial wear was observed in some appliances. Elasticity was qualitatively rated as preserved in all the appliances. No clinically apparent mucosal irritation, ulcerative lesions, or allergic reactions were recorded in any of the ten patients during follow-up. Patient comfort scores were higher at T6 compared with T0, and transient hypersalivation reported during the initial adaptation period resolved in all patients. **Conclusions:** Within the limitations of this prospective observational case series, the clinical findings indicate favorable appliance integrity and biological tolerance over six months of intraoral use. No clinically apparent adverse mucosal reactions were recorded. However, the qualitative nature of the physical assessments, the limited sample size, and the absence of instrumental testing do not allow definitive conclusions regarding material properties, comprehensive biocompatibility, or therapeutic effectiveness. Further studies with larger populations, longer follow-up, and objective laboratory assessments are warranted.

## 1. Introduction

Functional orthodontics has progressively evolved from a treatment philosophy primarily focused on dental alignment toward a comprehensive biological approach that considers the complex interaction between craniofacial growth, neuromuscular function, and dentofacial development [1]. Contemporary interceptive orthodontics recognizes that many malocclusions are not exclusively the consequence of abnormal dental positioning but rather the result of functional alterations involving tongue posture, orofacial musculature, swallowing patterns, and respiratory function [2]. Consequently, early orthodontic treatment increasingly aims not only to correct occlusal discrepancies but also to restore physiological function, thereby promoting harmonious craniofacial growth and improving long-term treatment stability [3].

Within this context, elastodontics has emerged as a minimally invasive therapeutic approach designed to exploit the biological plasticity of the growing patient [4]. Unlike conventional fixed appliances, which primarily produce tooth movement through continuous orthodontic forces, elastodontic appliances generate gentle functional stimuli that interact simultaneously with the dentition, the surrounding musculature, and the neuromuscular system. Their therapeutic action is therefore based on functional re-education rather than purely mechanical correction, encouraging the establishment of physiological oral functions capable of guiding dentoalveolar and skeletal development during growth [5].

Among the currently available elastodontic systems, AMCOP Bio-Activators have gained increasing attention in interceptive orthodontics because they combine orthopedic, orthodontic, and myofunctional effects within a single removable appliance [6]. Their clinical indications include Class II and Class III malocclusions, anterior open bite, deep bite, transverse discrepancies, dysfunctional swallowing, oral breathing, and abnormal tongue posture. By promoting a more physiological balance among the tongue, lips, cheeks, and masticatory muscles, these appliances seek to improve both occlusal relationships and overall stomatognathic function while taking advantage of the patient’s residual growth potential [7].

The therapeutic effectiveness of any elastodontic appliance, however, depends not only on its design and on patient compliance but also on the physical and biological characteristics of the biomaterial from which it is manufactured [8]. Because these devices remain in prolonged contact with oral tissues and are continuously exposed to saliva, thermal fluctuations, enzymatic activity, microbiota, cyclic mechanical loading, and variations in oral pH, the material must preserve its structural integrity, elasticity, resilience, dimensional stability, and surface characteristics throughout treatment. Even limited deterioration of these properties may compromise appliance performance, reduce patient comfort, and potentially influence treatment outcomes.

Equally important is the biological safety of the material. Devices intended for long-term intraoral use must demonstrate excellent biocompatibility, avoiding cytotoxicity, mucosal irritation, allergic reactions, or sensitization. Current European regulations and the ISO 10993 series establish standardized procedures for evaluating the biological safety of medical devices before commercialization [9]. According to the manufacturer’s product-specific documentation, the materials used in AMCOP appliances have undergone biological safety assessments in accordance with the applicable ISO 10993 requirements. The manufacturer also documents the absence of latex, bisphenol A (BPA), and phthalates. These product-related data should be distinguished from the clinical observations reported in the present study, which were limited to the assessment of oral tissue tolerance during routine appliance use.

Nevertheless, regulatory certification primarily reflects laboratory testing performed under controlled conditions, whereas the clinical behavior of the material during prolonged intraoral use may be influenced by the complex biological environment of the oral cavity.

Despite the growing clinical diffusion of elastodontic therapy, relatively few studies have specifically investigated the behavior of elastodontic biomaterials after extended clinical use. Most published reports focus on orthodontic outcomes such as occlusal correction, craniofacial growth, or functional improvement, whereas considerably less evidence is available regarding the maintenance of the material’s physical properties, its long-term biological tolerance, and its clinical durability. Consequently, information derived from laboratory certification should ideally be complemented by prospective clinical observations capable of documenting the actual performance of the appliance under routine therapeutic conditions [10].

For this reason, the present prospective observational study was designed to evaluate the physical behavior, biocompatibility, and biosafety of AMCOP elastodontic appliances during six months of routine clinical use. By prospectively monitoring ten consecutive patients undergoing interceptive orthodontic treatment, the study aimed to assess the maintenance of structural integrity, dimensional stability, elastic properties, surface characteristics, biological tolerance, patient comfort, and treatment compliance. The integration of standardized clinical observations with the manufacturer’s certified biological documentation may provide additional evidence regarding the reliability and safety of AMCOP elastodontic appliances for functional orthodontic therapy.

## 2. Materials and Methods

### 2.1. Study Design

This prospective observational study was conducted to evaluate the physical behavior, biocompatibility, and biosafety of AMCOP elastodontic appliances during routine interceptive orthodontic treatment. The investigation was carried out at the Department of Orthodontics of Policlinico di Bari and included ten consecutive growing patients treated according to the same standardized clinical protocol.

The study was designed as a real-world observational investigation without altering the routine therapeutic procedures or influencing the clinical management of the patients. Its primary purpose was to assess whether the physical and biological characteristics documented by the manufacturer could be confirmed after prolonged intraoral use under normal clinical conditions.

The AMCOP Bio-Activators used in this study were distributed by Micerium S.p.A. (Avegno, Genoa, Italy).

The study protocol was approved by the Independent Ethics Committee of the Azienda Ospedaliero-Universitaria Consorziale Policlinico of Bari, Italy, for the study entitled ‘Evaluation of the Effects of an Elastodontic Appliance in Patients with Dental and Skeletal Malocclusion’ (Protocol No. 7593).

Written informed consent was obtained from their parents or legal guardians before enrollment.

### 2.2. Study Population

Ten consecutive growing patients treated with AMCOP elastodontic appliances were included in the study (Table 1). All 10 patients initially evaluated completed the six-month follow-up and were included in the final analysis. No exclusions or losses to follow-up occurred. All ten patients screened were enrolled and included in the final analysis. No participants were excluded, lost to follow-up, or discontinued treatment. Furthermore, none of the appliances required replacement during the six-month observation period. The follow-up-related criteria were established to ensure complete clinical documentation and adherence to the study protocol; however, all enrolled participants fulfilled these requirements.

The study population therefore consisted of 10 patients, with complete follow-up data available for all participants.

The indication for treatment was established exclusively on the basis of routine orthodontic diagnosis and was entirely independent of the aims of the present investigation. Consequently, patient recruitment reflected normal clinical practice without any modification of the therapeutic decision-making process.

Additional inclusion criteria comprised regular attendance at all scheduled follow-up appointments, adherence to the prescribed appliance-wearing protocol, availability of complete clinical and photographic documentation, and written informed consent provided by the parents or legal guardians before enrollment.

Patients were excluded if they discontinued treatment before completion of the observation period, failed to comply with the prescribed therapeutic protocol, required replacement of the appliance because of accidental damage unrelated to normal physiological wear, or presented incomplete clinical records that prevented an adequate evaluation of the study outcomes.

### 2.3. Clinical Protocol

After appliance delivery, all patients received identical verbal and written instructions regarding appliance wear, maintenance, and oral hygiene procedures.

The prescribed protocol consisted of wearing the appliance for at least one hour during daytime and throughout the entire night, according to the standardized therapeutic protocol routinely adopted in our department.

To minimize variables that could influence material behavior, all patients followed the same maintenance protocol. The appliances were cleaned daily using Chanteclair Marseille neutral soap and thoroughly rinsed under running water before storage in the dedicated protective container. Compliance with both appliance wear and cleaning procedures was reinforced during every follow-up appointment to ensure protocol standardization throughout the study. Treatment compliance was assessed qualitatively during scheduled follow-up visits, based on the clinical follow-up and the information available regarding adherence to the prescribed wearing and cleaning instructions. No electronic monitoring or objective measurement of appliance-wearing time was performed. The categories “good” and “excellent” reflected the clinician’s qualitative assessment of reported adherence and were not derived from a validated quantitative compliance scale.

### 2.4. Clinical Assessment

Clinical evaluations were performed immediately after appliance delivery (T0) and repeated after six months of continuous use (T6). All examinations were carried out by the same experienced operator using standardized clinical criteria.

Baseline assessment confirmed the integrity of each appliance before intraoral use and verified the absence of manufacturing defects or structural abnormalities.

At the six-month follow-up, both physical and biological characteristics were systematically evaluated.

Dimensional stability was assessed qualitatively by clinical inspection of the appliance morphology and by checking for visible dimensional changes or permanent deformation after six months of use. Elastic behavior was assessed qualitatively during clinical examination by the same experienced operator. No instrumental measurements of dimensional changes or quantitative mechanical testing of elasticity were performed. Accordingly, these outcomes represent clinical observations rather than objective measurements of material properties.

Clinical evaluation focused on both the physical performance of the appliances and their biological compatibility throughout the six-month observation period. The physical assessment included a comprehensive examination of surface integrity, dimensional stability, preservation of the original morphology, maintenance of the material’s elastic properties, degree of superficial wear, presence of permanent deformation, occurrence of cracks or fractures, and preservation of overall clinical functionality. These parameters were selected to determine whether prolonged intraoral use could induce structural alterations potentially affecting the mechanical performance or therapeutic effectiveness of the appliances.

In parallel, biological compatibility was evaluated through clinical examination of the oral tissues and patient-reported outcomes. Particular attention was paid to the integrity of the oral mucosa, the presence of mucosal irritation, allergic reactions or signs of sensitization, as well as to patient comfort, which was assessed using a 10-point Visual Analogue Scale (VAS). Treatment compliance, the occurrence of transient hypersalivation during the adaptation phase, and overall patient satisfaction were also systematically recorded in order to provide a comprehensive evaluation of the clinical acceptability of the appliance. Patient-reported comfort was assessed using a 10-point Visual Analogue Scale (VAS), with 0 representing the lowest level of comfort and 10 representing the highest level of comfort. Higher scores therefore indicated greater comfort with the appliance.

All observations were collected using standardized clinical evaluation forms completed by the same examiner at each assessment. The recorded findings were subsequently organized into descriptive tables to facilitate comparison between baseline (T0) and the six-month follow-up (T6) and to provide a comprehensive overview of the physical and biological performance of the evaluated appliances.

Surface wear and overall physical condition were classified using a qualitative clinical assessment. Minimal wear referred to limited superficial changes, such as slight opacification or superficial wear, without visible cracks, fractures, permanent deformation, or loss of clinical functionality. Moderate wear referred to more noticeable superficial changes that remained confined to the surface and did not compromise structural continuity or clinical function. Overall physical condition was classified as excellent when no clinically relevant structural alterations were observed and as good when minor superficial changes were present without apparent impairment of appliance integrity or functionality. These categories were descriptive clinical classifications and were not based on quantitative thresholds.

### 2.5. Photographic Documentation

Standardized photographic documentation was obtained for every appliance after completion of the six-month observation period.

Images were acquired under identical photographic conditions using the same camera, lighting system, background, and magnification in order to ensure direct comparison among all evaluated appliances. For each subject, two standardized photographs were collected, consisting of a frontal view and an occlusal view.

Baseline photographic documentation was not included because all appliances were new at delivery and therefore presented identical physical characteristics. Consequently, photographic evaluation focused exclusively on the six-month follow-up, allowing qualitative assessment of surface wear, dimensional stability, preservation of morphology, and any clinically detectable structural alteration associated with routine intraoral use.

Due to space limitations and the considerable number of photographs obtained during the study, only two representative clinical cases are illustrated in the present manuscript. These examples were selected because they accurately reflect the overall findings observed across the entire study population. All ten appliances underwent the same standardized photographic evaluation after six months of clinical use, and the complete image dataset was included in the study analysis, although only representative images are presented here for clarity and conciseness.

### 2.6. Outcome Measures

The primary outcome of the study was the maintenance of the physical properties of AMCOP elastodontic appliances after six months of routine clinical use, with particular attention to dimensional stability, preservation of morphology, elastic behavior, surface integrity, and clinical functionality.

Secondary outcomes included the evaluation of biological tolerance, oral mucosal compatibility, patient-reported comfort, treatment compliance, adaptation time, transient hypersalivation, and overall patient satisfaction.

Considering the observational design and the relatively limited sample size, the study was intended as a descriptive clinical investigation. Therefore, no inferential statistical analyses were performed. Results are presented using descriptive clinical observations and qualitative comparisons of the evaluated variables.

## 3. Results

All ten patients completed the six-month follow-up according to the study protocol. No participant discontinued treatment, required appliance replacement, or experienced clinically relevant adverse events throughout the observation period. Attendance at all scheduled follow-up visits was complete, and compliance with the prescribed appliance-wearing protocol was considered satisfactory in every case, allowing a comprehensive clinical evaluation of both the physical and biological behavior of the appliances.

Baseline examination confirmed that all newly delivered AMCOP appliances exhibited identical physical characteristics before intraoral use, including optimal surface integrity, complete dimensional stability, preservation of the original morphology, optimal elasticity, and absence of structural defects. No manufacturing imperfections or abnormalities were detected during the initial clinical inspection.

**Figure 1:**
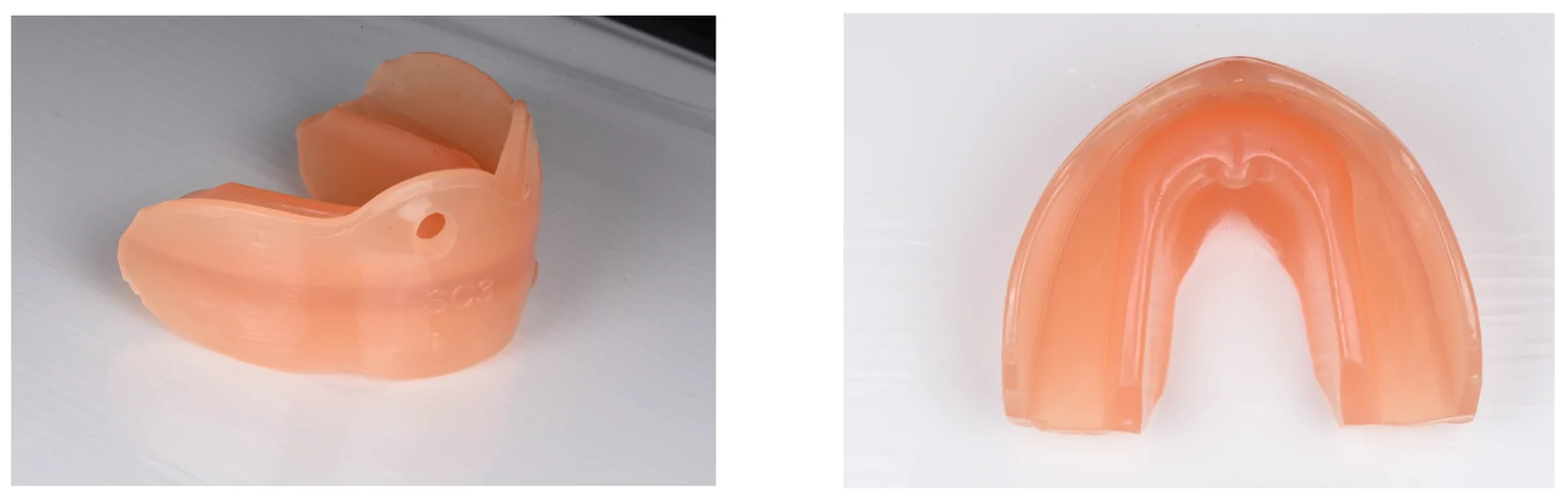
First clinical case: AMCOP bioactivator in lateral and occlusal view.

After six months of continuous clinical use, all appliances remained structurally intact and fully functional (Table 2). Dimensional stability was preserved in every appliance, and no permanent deformation, fractures, or clinically significant structural alterations were observed. The original morphology was completely maintained in most devices, whereas a limited number exhibited only minor physiological changes that did not interfere with appliance adaptation or therapeutic performance (Figure 1).

**Figure 2:**
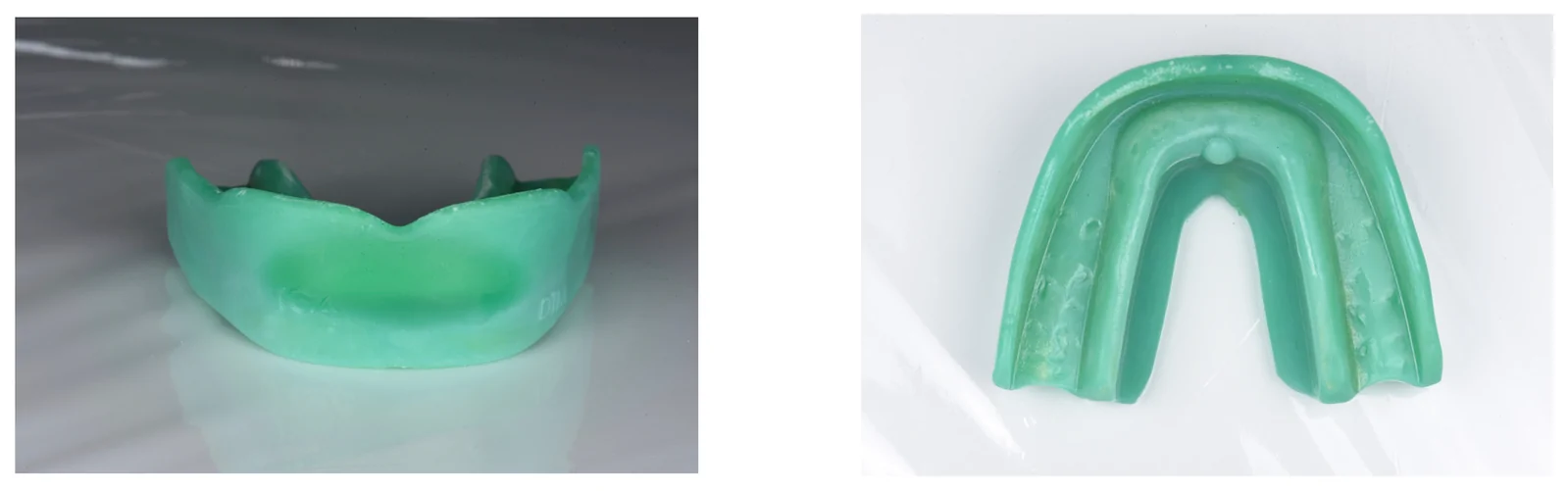
Second clinical case: AMCOP bioactivator in frontal and occlusal view.

Slight differences among individual appliances were observed with respect to superficial wear (Figure 2). Mild surface opacification and minimal wear were detected in several appliances, while two devices showed a slightly greater degree of physiological wear. Nevertheless, these findings remained limited to superficial changes and did not compromise structural continuity, elastic behavior, or clinical functionality. Elasticity was qualitatively rated as preserved in all the appliances. Overall clinical assessment classified five appliances as having an excellent physical condition and five as good, based on the qualitative evaluation of their surface integrity and structural appearance at T6.

The biological evaluation demonstrated equally favorable results (Table 3). At baseline, all patients presented healthy oral mucosa without signs of irritation, allergic reactions, or tissue sensitization. Initial comfort scores ranged from 6 to 8 on the Visual Analogue Scale (VAS), reflecting good acceptance of the appliance during the first phase of treatment. As expected for removable functional appliances, mild to moderate transient hypersalivation was reported during the first days following appliance delivery, representing a physiological adaptation response rather than an adverse biological event.

At the six-month follow-up, no clinically apparent mucosal irritation, ulcerative lesions, or allergic reactions were recorded in any of the ten patients. Transient hypersalivation reported during the initial adaptation period had resolved in all participants.

Patient-reported comfort scores were higher at T6 compared with T0, with final VAS scores ranging between 9 and 10 in all participants.

Compliance remained excellent or good across the study population, with adaptation times ranging from 8 to 15 days. Overall patient satisfaction was high, with most participants reporting that the appliance was comfortable to wear and easy to integrate into daily activities (Table 4).

Representative frontal and occlusal photographs obtained after six months supported the clinical observations by demonstrating preservation of appliance morphology and structural continuity despite minor physiological surface wear (Table 5). Collectively, the physical and biological findings indicate that AMCOP elastodontic appliances maintained adequate clinical performance throughout the observation period without evidence of clinically relevant material degradation or adverse biological reactions.

## 4. Discussion

The present prospective observational study was undertaken to investigate the physical behavior, biocompatibility, and biosafety of AMCOP elastodontic appliances under routine clinical conditions [11,12,13]. Although laboratory investigations and regulatory certification provide essential information regarding the composition and safety of orthodontic biomaterials, their actual clinical performance can only be confirmed through prolonged intraoral use [14,15,16,17,18,19,20]. The oral environment exposes removable appliances to continuous mechanical loading, temperature fluctuations, saliva, enzymatic activity, and repeated cleaning procedures, all of which may influence the long-term behavior of the material [21,22,23,24].

The findings of the present study indicate that AMCOP elastodontic appliances maintained their structural integrity and clinical functionality throughout six months of routine use [25,26,27,28,29,30]. No visible dimensional changes were observed on clinical examination in any of the ten appliances at T6. No permanent deformation, fractures, or clinically apparent structural discontinuities were detected [31,32,33,34,35]. Only minor physiological changes, consisting primarily of superficial polishing, limited opacification, and minimal wear, were observed in some appliances [36,37,38,39,40,41]. Importantly, these changes did not alter elasticity or compromise the therapeutic performance of the devices, suggesting that the material possesses sufficient mechanical stability to withstand the functional stresses encountered during everyday orthodontic treatment [42,43,44,45,46,47,48,49].

Maintenance of dimensional stability represents one of the most clinically relevant findings of this investigation [50,51,52,53,54]. Functional elastodontic appliances exert therapeutic effects through their specific morphology and elastic properties, which guide neuromuscular activity and dentofacial development [55,56,57,58,59,60,61]. Any clinically significant alteration of these characteristics could potentially reduce treatment effectiveness [62,63,64,65,66,67,68,69,70]. The absence of relevant structural modifications throughout the observation period therefore supports the mechanical reliability of the biomaterial during medium-term clinical use [71,72,73,74,75].

Equally encouraging were the biological outcomes [76]. No allergic reactions, persistent mucosal irritation, ulcerative lesions, or signs of tissue sensitization were recorded in any patient [77,78,79,80,81,82]. These clinical observations are distinct from the biological safety assessments documented by the manufacturer in accordance with ISO 10993. In the present study, no clinically apparent mucosal irritation, ulcerative lesions, or allergic reactions were recorded in the ten patients during the six-month follow-up. Although these findings are encouraging, they should not be interpreted as independent confirmation of comprehensive biocompatibility or biosafety [83,84,85,86,87,88,89,90]. Although clinical observation cannot replace laboratory biocompatibility testing, it represents an important complement because it reflects the behavior of the device under real clinical conditions [91,92,93,94,95,96].

Patient adaptation also followed the expected pattern observed with removable functional appliances [97,98,99,100,101,102,103]. Comfort scores were higher at T6 compared with T0, indicating improved patient-reported comfort at the final assessment. Treatment compliance was qualitatively rated as good or excellent, based on clinical follow-up and reported adherence to the prescribed wearing protocol [104,105,106,107,108,109]. The high levels of compliance observed throughout the study likely contributed to maintaining appliance integrity and may have positively influenced therapeutic effectiveness [110,111,112,113,114,115,116].

An important strength of this investigation is its prospective design and the standardized clinical protocol adopted for all participants [117]. Every patient received identical instructions regarding appliance wear and cleaning procedures, and all evaluations were performed by the same operator using predefined clinical criteria [118,119]. Furthermore, standardized photographic documentation provided additional qualitative evidence supporting preservation of appliance morphology after prolonged intraoral use [117,120].

Nevertheless, some limitations should be acknowledged. The study included a relatively limited number of patients and was conducted at a single clinical center [121]. In addition, the observation period was restricted to six months and therefore cannot provide information regarding long-term material aging throughout the entire duration of orthodontic treatment [122]. Finally, the investigation relied exclusively on clinical evaluation and did not include complementary laboratory analyses such as scanning electron microscopy, surface roughness measurements, mechanical testing, or chemical characterization after clinical use. Future studies integrating these objective laboratory techniques with prospective clinical observation would provide a more comprehensive understanding of elastodontic biomaterial performance.

## 5. Conclusions

Within the limitations of this prospective observational case series, the clinical findings indicate favorable structural integrity and clinical tolerance of AMCOP elastodontic appliances after six months of routine intraoral use. No visible dimensional changes were observed on clinical examination in any of the ten appliances. Elasticity was qualitatively rated as preserved in all the appliances. Furthermore, no clinically apparent mucosal irritation, ulcerative lesions, or allergic reactions were recorded in any of the ten patients during follow-up.

These findings provide preliminary clinical observations regarding appliance condition and patient tolerance under routine intraoral conditions. However, the qualitative nature of the assessments, the limited sample size, and the absence of instrumental testing do not allow definitive conclusions regarding the preservation of material properties, comprehensive biocompatibility, biosafety, or therapeutic effectiveness.

Further prospective studies involving larger populations, longer observation periods, and complementary instrumental and laboratory assessments are warranted to better characterize the long-term physical and biological behavior of elastodontic appliances.

## Figures and Tables

**Table 1:** Characteristics of the Patients Included in the Study.

Patient	Age	Sex	Initial Diagnosis	AMCOP Appliance	Observation Period
Case 1	8 years	M	Class II Division 1	SC2	6 months
Case 2	9 years	F	Class I	S2	6 months
Case 3	10 years	M	Class I	S3	6 months
Case 4	10 years	F	Class II Division 1	SC3	6 months
Case 5	12 years	M	OSAS	OSAS3	6 months
Case 6	10 years	F	Class III	DTM TC4	6 months
Case 7	9 years	M	Class I	DTM S3	6 months
Case 8	10 years	M	Class II Division 1	SC4	6 months
Case 9	11 years	F	Open bite	OPEN3	6 months
Case 10	9 years	M	Class I	OS3	6 months

**Table 2:** Physical characteristics of AMCOP appliances at baseline (T0).

Variable	Case 1	Case 2	Case 3	Case 4	Case 5	Case 6	Case 7	Case 8	Case 9	Case 10
Surface integrity	Excellent	Excellent	Excellent	Excellent	Excellent	Excellent	Excellent	Excellent	Excellent	Excellent
Dimensional stability	Complete	Complete	Complete	Complete	Complete	Complete	Complete	Complete	Complete	Complete
Shape preservation	Complete	Complete	Complete	Complete	Complete	Complete	Complete	Complete	Complete	Complete
Material elasticity	Optimal	Optimal	Optimal	Optimal	Optimal	Optimal	Optimal	Optimal	Optimal	Optimal
Surface appearance	Smooth	Smooth	Smooth	Smooth	Smooth	Smooth	Smooth	Smooth	Smooth	Smooth
Permanent deformation	Absent	Absent	Absent	Absent	Absent	Absent	Absent	Absent	Absent	Absent
Cracks/ Fractures	Absent	Absent	Absent	Absent	Absent	Absent	Absent	Absent	Absent	Absent
Clinical functionality	Optimal	Optimal	Optimal	Optimal	Optimal	Optimal	Optimal	Optimal	Optimal	Optimal

**Table 3:** Physical characteristics of AMCOP appliances after six months of clinical use.

Variable	Case 1	Case 2	Case 3	Case 4	Case 5	Case 6	Case 7	Case 8	Case 9	Case 10
Surface integrity	Excellent	Good	Excellent	Good	Excellent	Good	Excellent	Good	Excellent	Good
Dimensional stability	Complete	Complete	Complete	Complete	Complete	Complete	Complete	Complete	Complete	Complete
Shape preservation	Complete	Good	Complete	Complete	Complete	Good	Complete	Complete	Complete	Good
Material elasticity	Preserved	Preserved	Preserved	Preserved	Preserved	Preserved	Preserved	Preserved	Preserved	Preserved
Surface appearance	Smooth	Slightly opacified	Smooth	Slightly opacified	Smooth	Slightly opacified	Smooth	Slightly opacified	Smooth	Slightly opacified
Surface wear	Absent	Minimal	Minimal	Moderate	Absent	Minimal	Minimal	Minimal	Absent	Moderate
Permanent deformation	Absent	Absent	Absent	Absent	Absent	Absent	Absent	Absent	Absent	Absent
Cracks/ fractures	Absent	Absent	Absent	Absent	Absent	Absent	Absent	Absent	Absent	Absent
Clinical functionality	Unchanged	Unchanged	Unchanged	Unchanged	Unchanged	Unchanged	Unchanged	Unchanged	Unchanged	Unchanged
Overall clinical assessment	Excellent	Good	Excellent	Good	Excellent	Good	Excellent	Good	Excellent	Good

**Table 4:** Biological characteristics at baseline (T0).

Variable	Case 1	Case 2	Case 3	Case 4	Case 5	Case 6	Case 7	Case 8	Case 9	Case 10
Oral mucosal integrity	Intact	Intact	Intact	Intact	Intact	Intact	Intact	Intact	Intact	Intact
Mucosal irritation	Absent	Absent	Absent	Absent	Absent	Absent	Absent	Absent	Absent	Absent
Allergic reactions	Absent	Absent	Absent	Absent	Absent	Absent	Absent	Absent	Absent	Absent
Signs of sensitization	Absent	Absent	Absent	Absent	Absent	Absent	Absent	Absent	Absent	Absent
Initial comfort (VAS)	7	8	6	7	8	7	6	8	7	7
Transient hypersalivation	Moderate	Mild	Moderate	Moderate	Mild	Moderate	Mild	Moderate	Mild	Moderate
Expected compliance	Excellent	Good	Good	Excellent	Good	Excellent	Good	Excellent	Good	Excellent

**Table 5:** Biological characteristics after six months of clinical use (T6).

Variable	Case 1	Case 2	Case 3	Case 4	Case 5	Case 6	Case 7	Case 8	Case 9	Case 10
Oral mucosal integrity	Intact	Intact	Intact	Intact	Intact	Intact	Intact	Intact	Intact	Intact
Mucosal irritation	Absent	Absent	Absent	Absent	Absent	Absent	Absent	Absent	Absent	Absent
Allergic reactions	Absent	Absent	Absent	Absent	Absent	Absent	Absent	Absent	Absent	Absent
Signs of sensitization	Absent	Absent	Absent	Absent	Absent	Absent	Absent	Absent	Absent	Absent
Final comfort (VAS)	10	9	9	9	10	9	10	9	9	10
Treatment compliance	Excellent	Good	Good	Excellent	Excellent	Good	Excellent	Excellent	Good	Excellent
Transient hypersalivation	Absent	Absent	Absent	Absent	Absent	Absent	Absent	Absent	Absent	Absent
Adaptation time	10 days	8 days	15 days	12 days	9 days	11 days	13 days	8 days	10 days	14 days
Patient satisfaction	Very satisfied	Satisfied	Satisfied	Very satisfied	Very satisfied	Satisfied	Very satisfied	Satisfied	Very satisfied	Very satisfied

## Data Availability

The original contributions presented in this study are included in the article. Further inquiries can be directed to the corresponding author.

## Abbreviations

BPA	Bisphenol A
ISO	International Organization for Standardization
T0	Baseline (appliance delivery)
T6	Six-month follow-up
VAS	Visual Analogue Scale
